# A Dietary Intervention High in Green Leafy Vegetables Reduces Oxidative DNA Damage in Adults at Increased Risk of Colorectal Cancer: Biological Outcomes of the Randomized Controlled Meat and Three Greens (M3G) Feasibility Trial

**DOI:** 10.3390/nu13041220

**Published:** 2021-04-07

**Authors:** Andrew D. Frugé, Kristen S. Smith, Aaron J. Riviere, Rachel Tenpenny-Chigas, Wendy Demark-Wahnefried, Anna E. Arthur, William M. Murrah, William J. van der Pol, Shanese L. Jasper, Casey D. Morrow, Robert D. Arnold, Kimberly Braxton-Lloyd

**Affiliations:** 1 Department of Nutrition, Dietetics and Hospitality Management, Auburn University, Auburn, AL 36849, USA; kss0034@auburn.edu (K.S.S.); ajr0042@auburn.edu (A.J.R.); raychelynn_11@hotmail.com (R.T.-C.); 2 Department of Nutrition Sciences, University of Alabama at Birmingham, Birmingham, AL 35294, USA; demark@uab.edu; 3 Department of Food Science and Human Nutrition, Division of Nutritional Sciences, University of Illinois at Urbana-Champaign, Champaign, IL 61801, USA; aarthur@illinois.edu; 4 Department of Educational Foundations, Leadership, and Technology, Auburn University, Auburn, AL 36849, USA; wmm0017@auburn.edu; 5 Department of Computational Biology and Bioinformatics, University of Alabama at Birmingham, Birmingham, AL 35294, USA; liamvdp@uab.edu; 6 Department of Cell, Developmental and Integrative Biology, University of Alabama at Birmingham, Birmingham, AL 35294, USA; slj0012@auburn.edu; 7 Department of Drug Discovery and Development, Auburn University Harrison School of Pharmacy, Auburn, AL 36849, USA; caseym@uab.edu (C.D.M.); rda0007@auburn.edu (R.D.A.); 8 Department of Pharmacy Services, Auburn University Harrison School of Pharmacy, Auburn, AL 36849, USA; lloydkb@auburn.edu

**Keywords:** chemoprevention, colorectal cancer, diet, green leafy vegetables, red meat, 8-hydroxy-2′deoxyguanosine

## Abstract

Green leafy vegetables (GLV) may reduce the risk of red meat (RM)-induced colonic DNA damage and colorectal cancer (CRC). We previously reported the primary outcomes (feasibility) of a 12-week randomized controlled crossover trial in adults with habitual high RM and low GLV intake with body mass index (BMI) > 30 kg/m^2^ (NCT03582306). Herein, our objective was to report a priori secondary outcomes. Participants were recruited and enrolled in 2018, stratified by gender, and randomized to two arms: immediate intervention group (IG, n = 26) or delayed intervention group (DG, n = 24). During the 4 week intervention period, participants were provided with frozen GLV and counseled to consume 1 cooked cup equivalent daily. Participants consumed their normal diet for the remaining 8 weeks. At each of four study visits, anthropometrics, stool, and blood were taken. Overall, plasma Vitamin K1 (0.50 ± 1.18 ng/mL, *p* < 0.001) increased, while circulating 8OHdG (−8.52 ± 19.05 ng/mL, *p* < 0.001), fecal 8OHdG (−6.78 ± 34.86 ng/mL, *p* < 0.001), and TNFα (−16.95 ± 60.82 pg/mL, *p* < 0.001) decreased during the GLV intervention compared to control periods. Alpha diversity of fecal microbiota and relative abundance of major taxa did not differ systematically across study periods. Further investigation of the effects of increased GLV intake on CRC risk is warranted.

## 1. Introduction

The most recent global estimates of cancer incidence and mortality place colorectal cancer (CRC) as the fourth most prevalent and second deadliest cancer worldwide [1]. The World Cancer Research Fund International Continuous Update Project (CUP) scientists’ 2017 meta-analysis of 111 prospective cohort studies supported the relationships between increased risk of CRC with increased red and processed meat intake, as well as decreased risk of CRC with increased vegetable intake [2]. In 2015, approximately 38.3% of new CRC cases were directly attributed to suboptimal diets in the United States [3]. This high-meat, low-vegetable “Western” dietary pattern is most common in developed and developing countries and is directly associated with CRC risk [4], with a recent meta-analysis of 28 studies indicating a 30% increased risk of CRC for adults consuming this dietary pattern [5].

Excess adiposity has been recognized as another modifiable risk factor for colon cancer for more than two decades [6]. In a 2018 CUP meta-analysis of 47 cohort studies with 7,393,510 participants, increased body weight, body mass index (BMI), waist circumference, and waist-to-hip ratio were all independently associated with increased CRC risk in both men and women [7]. Since dietary behaviors are more easily improved than body composition [8], it is imperative to determine which dietary approaches and public health messages produce the greatest risk reduction. Based on our survey of 990 adults in the United States, a slight majority of respondents indicated they would not be willing to forego red meat (RM) consumption, and only 14.5% of men and 14.9% of women indicated that they did not like green leafy vegetables (GLV) [9]. Therefore, risk reduction may be more feasible via addition of GLV, rather than omission of RM.

A series of preclinical studies indicate that chlorophyll in GLV prevents the cytotoxic and carcinogenic effects of heme in RM [10,11,12,13], which is mediated by the microbiota residing in the colon [14,15]. 8-hydroxy-2′-deoxyguanosine (8-OHdG) is a marker of DNA damage associated with increased adenoma risk [16], which we aim to use as a proxy for cytotoxicity observed in preclinical models. High-sensitivity C-reactive protein (hsCRP) [17] is associated with elevated Proteobacteria [18] and has been linked with increased risk of colon cancer [19,20] and mortality [21]. Similarly, interleukin-6 (IL-6) has been implicated in colon cancer prognosis [22], metastasis [23], and mortality [24]. A 23-week plant-based diet (<50 g animal products/day) significantly reduced IL-6 and tumor necrosis factor-α (TNFα) in 89 obese adults [18], which corresponded with a decrease in Proteobacteria and an increase in *Bifidobacterium*. Since TNFα is produced primarily in response to lipopolysaccharide [25], a structural component of Gram-negative bacteria, it may also be a marker for mucosal health. In addition to the ability of chlorophyll to bind heme, it is hypothesized that the high flavonol content of GLV promotes the growth of several short-chain fatty acid-producing bacterial genera, which are associated with cytoprotective effects in the colon [26].

We sought to directly study the preliminary effects of increased GLV consumption in adults with increased BMI consuming a Western dietary pattern. The primary outcomes of this 12-week crossover trial were previously reported, indicating feasibility of accrual and retention, with adherence slightly below target but acceptable [27]. Herein, we report biological outcomes that may be relevant to CRC risk reduction, which include cytokines, gut microbiota, and Vitamin K1 as an objective measure of intervention adherence.

## 2. Materials and Methods 

### 2.1. Study Design and Aims

Detailed methods have been described previously [27]. The study was approved as protocol #18-180 EP 1806 by the Auburn University Institutional Review Board. It was conducted in accordance with the Declaration of Helsinki and pre-registered on ClinicalTrials.gov (NCT03582306). The aims of this report were determined a-priori and are included in the ClinicalTrials registry. The aim of this paper is to report all biological data relevant to oxidative DNA damage, inflammation, and microbiota. 

### 2.2. Participant Recruitment and Informed Consent

Participants were recruited via email from July to September 2018 in the Auburn-Opelika area in east Alabama. Interested individuals completed an online eligibility survey which included food frequency questionnaire questions to assess habitual RM and GLV consumption and were contacted for follow-up by study staff. Eligibility criteria were (1) current low-GLV consumption (<2 servings/day); (2) current high-RM consumption (>5 servings red meat/week); (3) high BMI (>30 kg/mP^2^ P); (4) willing to maintain normal prescription and/or supplement intake; (5) willing to adhere to dietary protocol; (6) ability to store and cook study foods; (7) English speaking and reading ability. Participants were excluded if they had a previous diagnosis of CRC or used oral or IV antibiotics, corticosteroids, immunosuppressive agents, or commercial probiotics within the last four weeks. Written informed consent was obtained prior to any post-screening data collection.

### 2.3. Randomization and Interventions

Randomization occurred after the completion of baseline assessment. Participants were stratified by gender into blocks of four, with each participant in successive order receiving the gender-specific envelope with the group assignment, which was generated by KSS [19]. Enrollment was conducted by KSS and ADF, and ADF assigned participants to intervention groups. All participants received the intervention in random order: either immediately (first four weeks of the 12-week study) or delayed (last four weeks of the 12-week study). During the intervention period, participants were provided with frozen GLV purchased by study staff directly from local retailers. Participants were given a recipe book and instructed to consume 1 cup cooked GLV daily (including spinach, kale, collards, mustard greens, and turnip greens). Additionally, they were encouraged not to alter any other elements of their diet, including red meat consumption. During the four-week washout and control periods, participants were asked to consume their habitual diet.

### 2.4. Data Collection

All biological measures were obtained at baseline and repeated every four weeks, with subjective measures obtained at each time point and reported previously [27]. Subjective measures included the Food Acceptability Questionnaire (FAQ) [28,29,30], Dietary Habits and Colon Cancer Beliefs Survey (DHCCBS) [9], the International Physical Activity Questionnaire (IPAQ) [31]. IPAQ data report frequency and duration of physical activity by intensity level and reports minutes sitting. Thus, we combined physical activity data to report total active minutes and total sitting minutes. Two 24 h dietary recalls were obtained at each timepoint by a dietetics student or Registered Dietitian. Recalls were entered by study staff into the Automated Self-Administered 24-Hour Dietary Assessment tool (ASA24) [32]. Calorie and macronutrient values reported for each time point represent the average of the two recalls obtained.

Height and weight were measured using standard procedures and used to calculate BMI; waist and hip circumferences were measured using a standard tape measure at each time point [33]. Body composition was analyzed using a handheld Body Impedance Analysis (BIA) instrument (Omron HBF-306C, Omron Healthcare, Inc. Lake Forest, IL, USA). 

Frozen fecal samples were obtained by study staff at each visit after participants collected them at their home using commode specimen collectors and sterile collection tubes and stored in their home freezers immediately. Patients reported the consistency of their stool using the Bristol Stool Forms Scale (BSFS) [34]. Samples were stored at −80 °C until further processing. Microbial genomic DNA was isolated using standard methods and kits from Zymo Research (Irvine, CA, USA), and the 250 base pair V4 region of the rRNA gene amplified by polymerase chain reaction and sequenced using the Illumina Miseq (San Diego, CA, USA). The informatic analyses were performed using the Quantitative Insight into Microbial Ecology (QIIME) suite, version 1.7 as modified by Kumar et al. (2014) [35,36]. 

Phlebotomy was performed by a trained phlebotomist; sera and plasma were separated in their respective collection tubes, aliquoted, and frozen at −80 °C until analysis. Oxidized guanine species (8-hydroxy-2′-deoxyguanosine [8-OHdG], 8-hydroxyguanosine, and 8-hydroxyguanine) were measured in fecal water to determine the genotoxicity of the lumen and concurrent oxidative stress in the plasma via enzyme-linked immunosorbent assay (ELISA) from StressMarq Biosciences (Victoria, Canada). To normalize fecal water concentrations, solid concentration was determined based on BSFS responses. BSFS responses fall into 7 types of stool, which is commonly compressed into 3 categories: 1: hard and lumpy (Types 1–2); 2: normal consistency (Types 3–5); and 3: loose and watery (Types 6–7) [37]. Previous research indicates stool classification type is correlated with water concentration, and estimated water content can be predicted with knowledge of stool type (1: 67%; 2: 72%; 3: 77%) [38]. Therefore, to determine solid concentration, we multiplied stool sample weight by the remaining percentage (out of 100%) and divided the calculated solid weight by total sample weight. This solid concentration value was then used to uniformly dilute samples for fecal water analyses. CRP and IL-6/TNFα were measured via ELISA kits from RayBiotech (Peachtree Corner, GA, USA) and ABCam (Cambridge, UK) respectively.

LC–MS/MS was used to analyze plasma vitamin K1. Phylloquinone (K1) and its deuterated internal standard, Vitamin K1-d7, were purchased from Sigma Aldrich (St. Louis, MO, USA) Stock solutions of the analyte and deuterated internal standard were prepared by dissolving each compound in MeOH: CH3Cl (2:1). Calibration and quality control (QC) samples were made from stock solutions and UV-treated (for vitamin K depletion), pooled plasma. The calibration range consisted of thirteen levels from 0.024–100 ng/mL. At the time of analysis, 300 µL aliquots of plasma were transferred to 1.8 mL polypropylene centrifuge tubes and spiked with 10 µL of the internal standard (30 ng/mL). Ice-cold acetonitrile (900 µL) was added to each tube and vortexed for fifteen seconds. Samples were then centrifuged at 1500 rpm for fifteen minutes at 4 °C, and the supernatant was subsequently transferred to glass vials. The samples were dried under a stream of N2 and reconstituted in 100 µL of MeOH: CH3Cl (2:1). Twelve microliters of the reconstituted solution was injected for analysis. An Agilent Technologies 1290 Infinity UPLC coupled via Agilent Jetstream electrospray ionization (AJ-ESI) to the 6460 Triple Quadrupole Mass Spectrometer (Agilent Technologies, Santa Clara, CA, USA) was used for the plasma analysis of vitamin K1. Samples were injected (2 µL) onto a reversed-phase Zorbax SB-C8 column, 1.8 µm, 2.1 × 50 mm (Agilent Technologies); the column temperature was kept at 40 °C. The mobile phase consisted of [A] 0.1% formic acid in 5mM NH4 formate and [B] 0.1% formic acid in methanol introduced at a flow rate of 0.5 mL/min. Chromatographic separation was achieved using gradient elution. The solvent composition was maintained at 70% [B] for the first 0.5 min, increased and held at 95% [B] from 1.5–10.5 min, and decreased back to 70% from 10.5–11 min. The AJ-ESI ion source was operated in positive ion mode, and the QQQ scan type used for analysis was multiple reaction monitoring (MRM). The transitions used for the quantification of vitamin K1 and internal standard vitamin K1-d7 were 451.3–186.95 and 458.4–194.1, respectively. Nitrogen was used as the drying gas (10 L/min at 350 °C), nebulizer (45 psi), and collision gas; the capillary voltage was set as 4500 V. The LLOQ for the method was determined as 2.9 pg. Intra- and inter-day accuracy and precision were assessed by analyzing six replicates of QC samples at the concentrations of 0.20, 3.13, and 50 ng/mL on three consecutive days. Calculated accuracy and precision within 15% (20% for LLOQ) were considered acceptable. 

### 2.5. Statistical Analysis

The primary outcomes were feasibility, which included accrual, retention, and adherence. Thus, power analysis was based on adherence. Setting alpha = 0.05, beta = 0.20, and n = 44 (assuming 10% attrition), 93% adherence would have resulted in a Cohen’s D = 0.80 [39]. Analysis of all biological outcomes were, therefore, exploratory and defined a priori at clinicaltrials.gov (https://clinicaltrials.gov/ct2/show/NCT03582306, accessed on 1 December 2020).

Statistical analyses were conducted in SPSS 24.0 (IBM Corp. Released 2016. IBM SPSS Statistics for Windows, Version 24.0. Armonk, NY, USA: IBM Corp.) with study arm allocation blinded to analysis. Descriptive statistics were obtained for study participants, who were compared using independent sample *t*-tests for continuous variables and chi-square tests for categorical variables. WMM conducted statistical analyses and was blinded to intervention assignment.

Treatment effects for biomarkers were analyzed with multi-variate analysis of covariance (MANCOVA) models which included fixed factors for treatment condition, study arm, period, and participant. Since participants were block-randomized by gender, this factor was also included in the models. Outcome measures that substantially deviated from normality were log-transformed prior to analysis. Statistical significance threshold was set at *p* = 0.05. Baseline measures at each period for the relevant biomarker measures were included as covariates to increase power and adjust for differences in pretreatment levels. In addition to accounting for the within-subjects design, the MANCOVA modeling also makes possible the simultaneous estimation of treatment effects and carryover effects. The latter was estimated by the coefficients for the study arm. Differences in measures at the beginning of each period across treatment conditions were assessed using *t*-tests. 

Microbiome analysis was conducted as described previously by Frugé et al. [40]. One-way Analysis of Variance (ANOVA) and Kruskal–Wallis tests adjusted with false discovery rates (FDR) were used to compare between-group and group x time differences in microbiota. 

## 3. Results

### 3.1. Study Participant Characteristics

Fifty participants were recruited and enrolled in the trial lasting from July 2018 to December 2018. The CONSORT diagram can be found in the 2019 report by Frugé et al. [27]. Baseline characteristics of study participants are shown in Table 1. Participants were mostly in their late forties and early fifties and had an average BMI of 36.2 (Class II obesity) and body fat percentage of 38.7. Based on food frequency questionnaire data from the study screener, participants consumed ten servings of red meat and less than one half a serving of green leafy vegetables weekly at baseline. Most participants were female (62%) and non-Hispanic White (80%). Randomization was only stratified by gender, and a larger proportion of African American participants were allocated to the delayed group (*p* = 0.035). Twenty-three out of 50 participants had graduate and/or professional degrees, and eight had an associate degree or lower, with the majority in the latter category being randomized to the delayed intervention. The majority (58%) of participants were married. In the first four weeks of the study, one participant withdrew consent and one participant was lost to follow-up after illness not related to the study—both were in the immediate intervention group. Forty-eight participants completed the study, forty of whom had complete biological sample data for analysis. Over the course of the study, no clinically or statistically significant changes were observed with regard to weight, BMI, and body fat percentage. 

At baseline, participants consumed 2083 ± 559 calories, coming from 86 ± 26 g protein, 93 ± 33 g fat, and 226 ± 75 g carbohydrate, with no differences in calories or macronutrients between groups. Additionally, no significant changes in total calories or any macronutrients were observed across time points. Participants reported an average total weekly active time of 928 ± 1494 min, with no significant changes across time points, though total active minutes trended downward over the course of the study (−230 ± 1048, *p* = 0.109). Total sitting time was 429 ± 195 for all participants, with the immediate group reporting more sitting time at baseline (502 ± 198 vs. 356 ± 165 min, *p* = 0.011). No significant differences in sitting time were observed across all other time points.

### 3.2. Circulating Biomarkers

Changes in circulating biomarkers are delineated in Table 2. On average, Vitamin K1 increased (*p* < 0.001) and plasma 8OHdG decreased (*p* < 0.001) during the intervention compared to control periods. Changes in the expected direction were observed in both groups, and these changes remained statistically significant in both the immediate and delayed intervention groups. Compared to the control period, fecal 8OHdG decreased during the intervention for the immediate (*p* < 0.001) and delayed (*p* < 0.001) groups. In the immediate group, TNFα decreased non-significantly, but reached significance in the delayed group during the intervention (*p* = 0.011) as well as compared to the control period (*p* < 0.001). In the delayed group, IL-6 increased significantly following the intervention compared to the control period (*p* < 0.001) but was not significantly altered in the immediate group (*p* = 0.242). No significant changes were observed for CRP.

Analysis of covariance tables for biological outcomes within participants are displayed in Table 3. Plasma 8OHdG significantly decreased between intervention and control periods, (*F*(1, 33) = 11.020, *p* = 0.002, ηp^2^ = 0.250). Statistical significance was maintained after controlling for intervention arm (*F*(1, 33) = 4.482, *p* = 0.042, ηp^2^ = 0.120) and baseline 8OHdG values (*F*(1, 33) = 8.077, *p* = 0.008, ηp^2^ = 0.197). Vitamin K1 levels differed significantly on treatment (*F*(1, 33) = 70.408, *p* < 0.001, ηp^2^ = 0.681).

### 3.3. Microbial Diversity and Taxa

**Figure 1:**
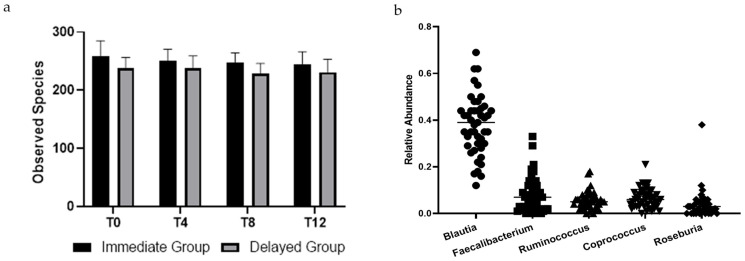
(**a**) Observed species of bacteria in stool samples collected from participants across all times points. There was no effect of time (*p* = 0.391) or group × time (*p* = 0.600). Points are means, and bars represent 95% confidence intervals. (**b**) Relative abundance of top five genera in stool samples collected at baseline of participants in a randomized controlled crossover high green leafy vegetable dietary intervention.

At baseline, no differences in alpha diversity were observed between groups. Over the course of each four-week period, no changes in alpha diversity beyond those expected due to sampling variability were observed for either group. Figure 1a reports the mean and 95% confidence interval for the observed species at each time point by group. 

**Figure 2:**
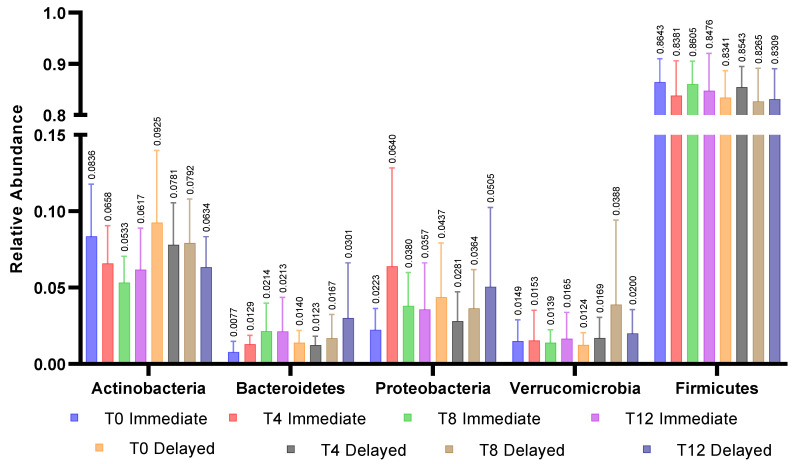
Relative abundance of 5 major phyla within groups across all time points. No significant differences were observed between the immediate and delayed groups within phyla or across timepoints within phyla.

There were no differences between groups in bacterial taxa at baseline. Five of the most prevalent genera were within the Firmicutes phylum and are reported in Figure 1b. Relative abundance at the phyla level for all participants were Firmicutes (84.7 ± 11.2%), Actinobacteria (8.9 ± 9.5%), Proteobacteria (3.4 ± 6.6%), Verrucomicrobia (1.4 ± 2.4%), and Bacteroidetes (1.1 ± 1.7%). Seven of the ten most abundant genera were Firmicutes. Baseline relative abundance of the five most abundant genera are reported in Figure 1a. Post-hoc Kruskal–Wallis tests compared operational taxonomic units (OTUs) between groups at each time point and within groups from pre- to post-intervention. Bray-Curtis dissimilarity tests with *p* < 0.05 were only observed between groups at the end of the first four-week period; no OTUs differed between groups after False Discovery Rate correction (Figure 2).

## 4. Discussion

This is the first trial to assess the effects of a dietary intervention high in GLV in adults with elevated BMI and high habitual RM consumption who are at increased risk for CRC. We report several often-cited biomarkers but capitalize on the novelty of measuring 8OHdG in both plasma and fecal water, the latter of which has not been reported to date. While our primary outcomes report noted subjective increases in GLV and Vitamin K consumption, we additionally measured plasma Vitamin K1 as an objective measure of adherence to the protocol. Finally, we report on the microbial structure of stool samples and the (lack of) changes to the microbiome over the course of the 12-week study. 

It is imperative to note that Vitamin K1 analyses indicate that there was a significant drop-in for participants during the control periods (i.e., participants continued or began consumption of GLV off protocol). The raw data suggest that five participants in the immediate group continued high GLV consumption throughout the entire study (anecdotally, reports of decreased indigestion and improved gastrointestinal function led participants to continue their newly formed GLV habit) and ten subjects in the delayed group voluntarily began consuming GLV either prior to or during the first four weeks of the study. A shortcoming in our study implementation was that we did not tell participants to avoid GLV during the recruitment and consent process. Nonetheless, the treatment*arm interaction for Vitamin K1 and 8OHdG is significant because the delayed group was also less adherent during the intervention period and did not experience the significant changes in Vitamin K1 and 8OHdG that were observed in the immediate group. Therefore, the effects of the intervention on biomarkers were likely diminished by non-adherence to the protocol.

Vitamin K1 was above detectable limits in only eight participants at the initial study visit, indicating that our screening method assessing habitual GLV intake was reliable. The significant increase in Vitamin K1 during the intervention for both immediate and delayed groups suggests meaningful adherence to the protocol. A weeklong intervention providing 100 g broccoli daily reported a two-fold increase in plasma Vitamin K1 [41]. In our study, seven of 18 objectively adherent participants in the immediate group had undetectable Vitamin K1 levels after the 4-week washout period. Given the observation that Vitamin K1 levels can be increased in seven days and diminished in 28 days or less, it is clear that the potential benefits of GLV might require sustained consumption. Estimating a minimally therapeutic dose of GLV is warranted, though it is already established that a number of genetic variants affect Vitamin K1 absorption and metabolism [42].

Several DNA damage metabolites including 8OHdG have been measured, most prominently in urine, for decades. Since plasma or urinary levels of these metabolites are heavily influenced by kidney function and excretion, these measures have limited value as a cross-sectional screening tool [43]. Conversely, plasma 8OHdG is a promising biomarker in assessing longitudinal exposure to oxidative stress with relevance for cancer prevention and control [44] given its relevance to mutagen formation and carcinogenesis [45]. 

Few diets and dietary supplement interventions have assessed changes in 8OHdG in urine and even fewer in plasma. Urinary 8OHdG was decreased (*p* = 0.041) after 4 weeks of agraz (berry juice/nectar, 200 mL) supplementation in 40 women with metabolic syndrome participating in a crossover trial [46]. In a study of 50 men and women with metabolic syndrome randomized to receive 30 g mixed nuts daily for 12 weeks vs. control, urine 8-oxo-7-hydro-2′-deoxyguanosine (8-oxodG) decreased significantly more in the intervention group (−2.42 nmol/mmol creatinine, *p* < 0.001) compared to the control group [47]. Though we did not assess blood pressure or insulin sensitivity to determine metabolic syndrome in our study participants, age and BMI were comparable to participants in both of these studies.

An 8-week vegetable (300 g) and polyunsaturated oil (25 mL) supplemented dietary intervention did not decrease urinary 8-oxodG and 8-oxo-7,8-dihydroguanosine (8-oxoGuo); however, DNA damage assessed through peripheral blood mononuclear cells indicated lower double-stranded DNA breaks in 54 patients with diabetes [48]. The only comparable study assessing plasma 8OHdG was in postmenopausal women (n = 48) receiving 22 g blueberry powder daily vs. placebo, in which investigators observed a decrease in plasma 8OHdG at four (*p* = 0.04) but not eight weeks [49]. In addition to Vitamin K1, GLVs contain glucosinolates, carotenoids, folate, and other DNA-protective compounds [50]. Since all of the above-cited interventions contained phytochemical- and antioxidant-rich foods and supplements, the reduction in oxidative DNA damage observed in our study could also be expected.

In designing this clinical trial, we hypothesized that the dietary fiber in GLV would support the function and integrity of the intestinal epithelium, which would be mediated by bacteria and result in decreased systemic inflammation [51]. In short, lipopolysaccharide from Gram-negative bacteria activate Toll-like receptor 4, increasing intestinal permeability, immune activation, and production of TNFα [25,52]. While CRP is a non-specific cytokine, a recent meta-analysis observed that increased CRP is associated with risk of CRC [19]; elevated IL-6 has been associated with CRC recurrence [22].

These cytokines have been measured in numerous diet and dietary-supplement interventions aimed primarily at cardiometabolic and diabetes-related outcomes. Relevant studies typically observe decreases in one or two, but rarely all three of these cytokines. These interventions include grape and grapeseed extracts, tart cherry juice, and avocado, as well as supplements aimed to modulate microbiota using synbiotics, kefir, and resistant starch [53,54,55,56,57,58,59,60,61,62]. Our observed trend in TNFα reduction may be spurious given the relative stability of the gut microbiota during the 12-week study.

Though there were statistically significant changes in some of these biomarkers, minimum clinically important difference (MCID) for prevention of CRC have not been established. Associations between risk, progression, and/or mortality of CRC with these biomarkers have been observed and can inform but not validate MCID at this time. Thus, we propose the following MCIDs: plasma 8OHdG—11 ng/mL [16], serum TNFa—30 pg/mL [63], serum IL6—3 pg/mL [63], serum CRP—1750 pg/mL [64]. Plasma 8OHdG was the only biomarker in our study that had changes close to the proposed MCID, but longitudinal studies are needed to validate each of these. 

The relative abundance of Firmicutes in our sample is higher than our lab has previously observed in overweight and obese men and women in the southeastern U.S. [40,65]. Since alpha diversity measured by observed species was also lower, it is unlikely that a potential overgrowth of species after collection caused an increase in relative abundance of Firmicutes. Nonetheless, we hypothesized the exponential increase in dietary fiber and bioactive plant compounds from GLV during the intervention period would increase microbial diversity [66]; however, diversity remained relatively constant for all participants throughout our study.

Three of the top five genera (Figure 2) are known butyrate producers; within these genera are species in Clostridium Clusters IV and XIVa [67]. Increased abundance of these bacteria that convert otherwise unabsorbable carbohydrates (soluble fibers, resistant starches, etc.) into absorbable short-chain fatty acids result in greater amounts of energy harvested from food by the host [68] and may contribute to obesity [69]. More specifically, species within the *Faecalibacterium, Ruminoccocus,* and *Roseburia* genera produce butyrate, which is beneficial to epithelial cells; however, the healthy phenotype results in a marked increase in Bacteroidetes [70], which was not observed in our participants. Recently, abundance of *Blautia* has been inversely associated with visceral fat area in both men and women [71]. Posthoc analysis of our data did not support this finding, possibly because our study population had BMI > 30, leaving no lean comparators. 

### Limitations

Because participants were recruited primarily from within the faculty and staff of a university in the southeastern United States, adults with a bachelor’s degree or higher were overrepresented (48%) compared to the national (31.5%) and state (24.9%) averages [72,73]. Twenty percent of participants were African American, which is lower than the state (26.8%) but higher than the national average (13.4%) [72,73]. Thus, adherence and effects of the intervention may not be generalizable to other populations.

Though the crossover design in this study allowed us to maximize potential effects given our modest sample size, the high rate of drop in both prior to and after intervention periods diluted many of the trends, which did not reach statistical significance in biomarkers. A brief pre-trial run-in control period may have prevented the initial drop-in by some and allowed more time to educate participants on the value of strictly adhering to protocol not only during the intervention but in the control period as well. Since we were the first group to report fecal 8OHdG, our methods may not have been sensitive enough to adjust for the water content of original samples; thus, these results should be interpreted with caution. Nonetheless, though not powered to detect changes in biomarkers, plasma and fecal 8OHdG as well as serum TNFα were significantly decreased by the intervention, which warrants further investigation.

## 5. Conclusions

This randomized controlled crossover dietary intervention is the first to report potential benefits of increasing green leafy vegetable consumption in adults at increased risk for CRC. Because of the small sample size resulting from powering the study for feasibility, the results are exploratory and should be interpreted with caution. Nonetheless, plasma and fecal 8OHdG, a biomarker of DNA damage, and serum TNFα were decreased by the intervention in conjunction with increased plasma Vitamin K1, the objective measure of dietary adherence. The direction of key effects and the heterogeneity of the effects evidence across individuals suggest that a larger study is warranted. Additionally, it is important to determine whether this can be replicated in a larger, more diverse population and to further explore the relationship between decreased 8OHdG and CRC risk reduction. 

## Figures and Tables

**Table 1:** Characteristics of participants in a randomized controlled crossover high green leafy vegetable dietary intervention.

	Total	Immediate	Delayed	
	(*n* = 50)	(*n* = 26)	(*n* = 24)	
	-------- Mean (SD) --------			*P*
Age (years)	48 (13.1)	47 (13)	49 (13)	0.649
Body Mass Index (kg/m^2^)	36.2 (4.7)	35.2 (4.6)	37.3 (4.8)	0.123
RM servings per week	10.3 (5.0)	10.5 (4.8)	10.2 (5.4)	0.846
GLV servings per week	0.21 (0.25)	0.20 (0.26)	0.22 (0.23)	0.852
	-------- *N* (%) --------			*P*
Gender				1.000
Male	19 (38)	10 (39)	9 (38)	
Female	31 (62)	16 (61)	15 (62)	
Race				0.035
African American	10 (20)	2 (8)	8 (33)	
White	40 (80)	24 (92)	16 (67)	
Education				0.050
Associate degree or less	8 (16)	1 (4)	7 (29)	
Bachelor’s degree	19 (38)	11 (42)	8 (33)	
Advanced degree(s)	23 (46)	14 (54)	9 (38)	
Marital Status				0.775
Married	29 (58)	16 (62)	13 (54)	
Not currently Married	21 (42)	10 (38)	11 (46)	

**Table 2:** Changes in circulating biomarkers of participants in a randomized controlled crossover high green leafy vegetable dietary intervention.

	Baseline	Intervention Change		Control Change		
All participants (*n* = 40)	Mean (SD)	Mean (SD)	*p*-value ¹	Mean (SD)	*p*-value ¹	*p*-value ^2^
Vitamin K1 (ng/mL)	0.1 (0.27)	0.48 (0.8)	0.0005	0.04 (0.72)	0.757	<0.001
8OHdG (ng/mL)	41.81 (18.18)	−8.05 (14.11)	0.001	1.25 (11.5)	0.507	<0.001
Fecal 8OHdG (µg/mL)	24.31 (54.52)	−12.06 (39.66)	0.040	−5.29 (29.41)	0.219	<0.001
TNFa (pg/mL)	156.15 (43.5)	−22.49 (47.41)	0.005	−5.21 (35.31)	0.369	<0.001
IL6 (pg/mL)	5.07 (3.17)	0.97 (3.46)	0.083	0.9 (5.13)	0.285	<0.001
CRP (pg/mL)	3251 (3965)	870 (3884)	0.926	−601 (3679)	0.321	0.945
Immediate Group (*n* = 21)						
Vitamin K1 (ng/mL)	0.06 (0.18)	0.79 (0.97)	0.001	−0.10 (0.75)	0.550	0.004
8OHdG (ng/mL)	45.56 (22.02)	−11.23 (16.25)	0.005	4.74 (12.18)	0.090	0.003
Fecal 8OHdG (µg/mL)	38.33 (73.85)	−24.92 (53.23)	0.031	−6.41 (39.24)	0.432	<0.001
TNFa (pg/mL)	166.48 (56.68)	−22.54 (57.58)	0.088	−14.85 (41.51)	0.117	0.203
IL6 (pg/mL)	4.56 (2.09)	0.7 (3.67)	0.395	1.42 (3.06)	0.046	0.242
CRP (pg/mL)	3543 (4657)	−204 (4981)	0.853	−1249 (4516)	0.220	0.922
Delayed Group (*n* = 19)						
Vitamin K1 (ng/mL)	0.15 (0.34)	0.14 (0.33)	0.072	0.20 (0.67)	0.231	<0.001
8OHdG (ng/mL)	36.95 (10.2)	−4.54 (10.64)	0.079	-3.06 (9.2)	0.189	<0.001
Fecal 8OHdG (µg/mL)	10.29 (14.51)	0.8 (5.92)	0.514	-4.16 (14.99)	0.187	<0.001
TNFa (pg/mL)	143.53 (8.1)	−22.42 (34.42)	0.011	6.69 (21.36)	0.215	<0.001
IL6 (pg/mL)	5.68 (4.11)	1.28 (3.27)	0.106	0.26 (6.95)	0.877	<0.001
CRP (pg/mL)	2893 (3005)	101 (2599)	0.867	200 (2136)	0.704	0.902

^1^ within-group change during the 4-week period; ^2^ within-group comparison between intervention and control periods using normalized data.

**Table 3:** Analysis of Covariance results for biological outcomes within participants in a randomized controlled crossover high green leafy vegetable dietary intervention.

Variables#	F	*p*-Value	ηp^2^
**Vitamin K1**			
Treatment	70.408	<0.001	0.681
Treatment*Gender	1.239	0.274	0.036
Treatment*Pre-Intervention Vitamin K1	0.468	0.499	0.014
Treatment*Arm	9.055	0.005	0.215
**8OHdG**			
Treatment	11.020	0.002	0.250
Treatment*Gender	1.462	0.235	0.042
Treatment*Pre-Intervention 8OHdG	8.077	0.008	0.197
Treatment*Arm	4.482	0.042	0.120
**Fecal 8OHdG**			
Treatment	2.256	0.142	0.061
Treatment*Gender	1.894	0.177	0.051
Treatment*Pre-Intervention Fecal 8OHdG	0.780	0.383	0.022
Treatment*Arm	2.550	0.119	0.068
**TNFa**			
Treatment	13.713	0.001	0.294
Treatment*Gender	0.000	0.985	0.000
Treatment*Pre-Intervention TNFa	12.281	0.001	0.271
Treatment*Arm	6.629	0.015	0.167
**IL6**			
Treatment	8.897	0.005	0.212
Treatment*Gender	0.185	0.670	0.006
Treatment*Pre-Intervention IL6	0.191	0.665	0.006
Treatment*Arm	4.299	0.046	0.115
**CRP**			
Treatment	1.513	0.227	0.044
Treatment*Gender	1.119	0.298	0.033
Treatment*Pre-Intervention CRP	1.625	0.211	0.047
Treatment*Arm	0.036	0.850	0.001

## Data Availability

Deidentified data may be obtained from the corresponding author upon written request.
